# Vesicoureteral Reflux in the Child with Lazy Bladder Syndrome: The Infrequent Voider

**DOI:** 10.1155/2008/432576

**Published:** 2008-07-03

**Authors:** Marco Grasso, Fabrizio Torelli, Salvatore Blanco, Flavio Fortuna, Marco Baruffi

**Affiliations:** Urology Department, Desio Hospital, 20033 Desio, Milan, Italy

## Abstract

The Infrequent Voider Syndrome or Lazy Bladder Syndrome in children is characterized by a large capacity bladder, frequently associated with a significant volume of residual urine. Usually these patients arrive at medical examination with a history of recurrent urinary infections but without anomalies in the upper urinary tract. We report about a young girl affected by one-sided 2° degree vesico-ureteral reflux due to Lazy Bladder Syndrome that had never been diagnosed before. This patient has been submitted to a prompt bladder training and seems presently to have at last gained a physiological micturition after 9 months of follow-up, without actual evidence of vesicoureteral reflux. Therefore we must stress that it is prominently important considering about infrequent micturition in a paediatric case history or a large capacity bladder, possible presence of bladder dysfunction and vesicoureteral reflux too.

## 1. INTRODUCTION

It has been known for nearly 30 years that there are children (mainly girls) who acquire abnormal micturition patterns in their first years of life, even without neurological or urological injuries. Such anomalies may lead to a sheer bladder disorder. Every child that is affected by altered bladder dynamics is at risk of developing pathologies such as urinary infection, vesicoureteral reflux (VUR) and so on.

We report about “a classic” case of Infrequent Voider Syndrome that we observed after the patient wandered for a long time across several other urologic and paediatric services. This is an evidence about how such a clinical picture has not an easy diagnosis and is often ignored.

## 2. CASE REPORT

About 2 years ago a 5-year-old girl with recurrent urinary infection was admitted in another hospital and checked. A 2° grade active right vesicoureteral reflux was found by voiding cystourethrography. She was treated by assuming orally a long-term antibacterial therapy: a prophylactic evening dose of trimethoprim-sulfa combination. After one year vesicoureteral reflux remained unchanged by X-ray check and she had a urinary infection relapse for a temporary break in antibacterial therapy. The parents took her to another urological department and were advised to submit their daughter to ureteral reimplantation surgery.

They were not persuaded and took their little girl to our outpatients' department.

**Figure 1:**
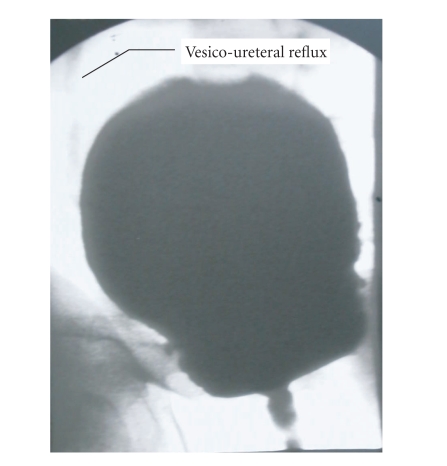
Large vesical capacity and vesicoureteral reflux at cystourethrography.

She was a little girl presenting normal somatic characteristic for her age, quite shy and affectively deeply attached and dependent from her mother who accompanied her. Her physical objectivity did not signal anything which could lead to urological pathologies. Looking at the cystourethrography (Figure 1) we observed a large vesical capacity, abnormal for the patient's age; an ultrasound exam of the urinary tract confirmed this observation: her bladder contained a volume of 500 cc.

The girl seemed calm with no need of urinating. Finally, persuaded by her mother she had an uroflowmetry test; the girl completely emptied her bladder, urinated 500 mL, with a normal flow.

Searching in the remote pathological anamnesis we found that the girl had always urinated rarely, twice or three times a day, remaining sometimes for more than 12 hours without urinating.

She had often refused to urinate in the evening before going to sleep, saying she did not need to.

Her mother did not attribute any importance to this habit and she did not insist. In both of them it was clear a fear of urinating in bathrooms out of their home because of the scarce hygiene conditions and the past urinary infections of the girl.

The patient refused every kind of invasive diagnostic approach such as urodynamic examination, cystoscopy. We could proceed in the diagnosis only by asking a micturition diary which has confirmed our clinical suspect of Lazy Bladder Syndrome.

From a therapeutic point of view, the girl has been directed to a bladder training therapy.

We only used behavioral therapy notifying to the girl a strict daily micturition scheme to modify the child's voiding habit and achieve a different behavior which included more frequent micturition and a new conception of the use of public toilettes. Consulting did not prescribe any other approach considering the good level of cooperation of the girl.

The absence of evident postmicturition residuals enabled us to avoid anticolinergic-alpha-adrenergic blockade therapies or invasive procedures such as intermittent catheterization or endovesical electrical stimulation.

After 9-month follow-up the patient seems to have changed her micturition habits; she is still following a prophylactic antibacterical therapy.

The last ultrasonographic evaluation does not show any negative evidence of the upper urinary tract.

The cystography check done 9 months later than the previous one and after 6 months of behavioral therapy pointed out the disappearance of the vesicouretheral reflux.

## 3. DISCUSSION

The Lazy Bladder Syndrome mostly concerns young girls. The history usually starts at 5–10 years of age with urinary infections, daytime incontinence, or as an accidental report during a consulting for other purposes.

Typically the bladder is expanse, easily palpable as it can have a urine content of more than 1 litre, often with a conspicuous postmicturition residual and surprisingly a normal upper urinary system.

The cause is unknown, but it probably has a behavioral origin. It regards children who learned to retain the urine for long periods. It is not uncommon that their parents inculcated them, even if not intentionally, the fear of contaminate themselves or even the idea that it is “evil” getting wet of urine.

It is often found in children excessively tidy or clean which makes them avoid any toilette that is not their own one.

They often urinate only in the morning and in the evening to avoid the school bathrooms.

There are some other children who had in their first years painful micturition because of urinary infections, and they still have a deep fear of it, getting used to urinate as less as possible [1].

Next to these clear clinical cases, we can find less evident ones in which we can find the absence of postmicturition residual but always high capacity bladders, as in our case report.

The uroflowmetry test can appear morphologically normal, making it harder to recognize a latent bladder disfunction, which can lead, if not treated, to irreparable damages of the upper urinary tract (Hoebeke et al. found in these children 17% VUR and Njman 20%) [2, 3]. Therefore, the risk run by our little 4-year-old patient was very high.

An urodynamic evaluation may prove, in advanced cases, detrusor hypocontractility from the permanent bladder iperextension and the need of a quite strong abdominal effort, depending on the coexistence of vesical sphincter dyssynergy, therefore a high pressure bladder emptying.

It is possible that in some cases, this determines and prevents the spontaneous resolution of a ureteral reflux, as in our case report, considering the correlation between reflux, capacity, and bladder pressure.

In these cases, the rieducative treatment of bladder training becomes necessary to lead the child to urinate psychologically without effort, without bladder iperextension trying to recover the vesicoureteral junction competence and a normal bladder volume. In fact, it is very difficult to gain some advantages when voiding disorders have already caused important alteration of urinary apparate. It is arduous to differentiate between lazy bladder and neurological bladder syndrome.

This is why an early diagnosis can be more advantageous than ever and we believe that the finding of a high bladder capacity in the child with infrequent micturition has to be always considered with worry and accurately evaluated, as a possible clinical expression of Lazy Bladder Syndrome.

To sum up, always for the purpose of an early diagnosis, we propose a diagnostic algorithm (Algorithm 1).

The management of vesicoureteral reflux (VUR) should be performed after a careful diagnostic approach. It is important to remember that high-bladder pressures may induce VUR. Voiding frequency, bladder capacity, and residual urine volume are key points facing children with recurrent urinary tract infections (UTIs).

## Figures and Tables

**Algorithm 1:**
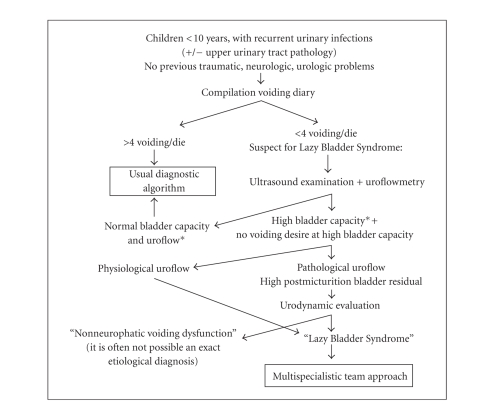
Diagnostic algorithm for Lazy Bladder Syndrome (*Bladder Capacity = age (years) + 2 × 30 mL).
